# Evaluating the impact of a pilot programme for home- and community-based services on long-term care needs among older adults in China

**DOI:** 10.1371/journal.pone.0311616

**Published:** 2024-11-21

**Authors:** Ruru Ping, Bo Hu, Takashi Oshio

**Affiliations:** 1 Hitotsubashi Institute for Advanced Study, Hitotsubashi University, Tokyo, Japan; 2 Care Policy and Evaluation Centre, Department of Health Policy, London School of Economics and Political Sciences, London, United Kingdom; 3 Institute of Economic Research, Hitotsubashi University, Tokyo, Japan; Victoria University, AUSTRALIA

## Abstract

China is facing escalating demand for long-term care (LTC), prompting the central government to launch a 5-year pilot programme to strengthen home- and community-based services (HCBS) from 2016 to 2020. However, the effects of this pilot programme on LTC needs remain unknown. This study aimed to evaluate the programme’s impact on LTC needs of older adults. Using panel data from the China Health and Retirement Longitudinal Study (*N* = 3,327), we evaluated the effects of the programme using a combined approach of propensity score matching and difference-in-differences. We found that the implementation of the HCBS programme reduced the number of unmet needs for assistance with instrumental activities of daily living (IADL) and delayed the progression of IADL limitations among older adults. However, no significant impact was found on the number of unmet needs for assistance with activities of daily living (ADL) or the progression of ADL limitations. Heterogeneity analysis showed that the effects of the HCBS programme on ADL and IADL limitations were weaker among those living in the Eastern region, and the effects on ADL limitations were stronger among those living with other people in the same household. Our findings suggest that implementing the HCBS programme can effectively address unmet care needs and delay the decline in functional capability among older adults. However, special attention should be paid to older adults living alone to mitigate the inequalities in functional limitations.

## Introduction

Spurred by a rapidly ageing population, China faces a high yet escalating demand for long-term care (LTC) services. According to the Seventh National Census, people aged 65 years and over accounted for 13.5% of the total population in 2020 [1], and this percentage is projected to reach 30% by 2050 based on the World Population Prospect 2022 [2]. Consequently, the number of older people requiring LTC is estimated to increase by 14.02 million by 2030, up from 102.24 million in 2020 [3]. The Chinese central government envisions home- and community-based services (HCBS) as the foundation of China’s LTC system and promotes a ‘90–7–3’ framework for aged care. This framework suggests that 90% of the older adults receive home-based or informal care, 7% receive community care, and 3% receive institutional care. The expenditure on HCBS is projected to increase from 0.7% of the gross domestic product (GDP) in 2020 to 6.4% by 2050 [4]. Providing accessible, affordable, and high-quality LTC, particularly HCBS, to older adults has become a pressing social issue in China.

Although many older adults in China prefer to age in place, the HCBS sector remains underdeveloped compared to institutional care and is concentrated in urban areas [5]. Developing HCBS in rural areas poses significant challenges owing to inadequate resources, insufficient policy attention, and widespread geographical dispersion of residents [5]. In large cities, government subsidies incentivise community centres to establish a small number of beds for older people with disabilities in need of overnight or short-term stays [5]. Overall, the availability of HCBS is low in China and is particularly inadequate in rural areas. By 2014, community-based adult day care centers covered approximately 50% of urban communities and only 20% of rural villages [6].

Despite these challenges, HCBS plays a crucial role in supporting the health and wellbeing of older adults. Verbrugge and Jette [7], in their Disablement Process Model, argued that providing personal assistance to individuals with functional limitations can enable them to function adequately while slowing the progression of disablement. Evidence from Western countries indicates that the use of HCBS is beneficial in reducing the risk of institutionalisation and improving the physical and mental health of older adults, particularly under conditions of high levels of stressors [8, 9]. This is observed in situations such as older unmarried women with functional limitations, older minorities facing stressful life situations, and older community dwellers experiencing moderate loneliness [10–12]. It is suggested that using HCBS helps older adults maintain their social networks, thereby enhancing their health and wellbeing [8].

The limited availability and unequal development of HCBS in China pose great challenges to meeting the increasingly diverse needs of older people. The existing studies show that the young-old residing in urban areas, especially in Eastern China, express a strong demand for community-based care services [13]. Conversely, individuals with severe functional limitations, low-income status, or those living in rural areas have shown a significant demand for home-based medical or nursing services [13]. Those without functional limitations or with a high-income status prefer social activities or entertainment services [13]. Therefore, it is crucial to address these diverse needs by strengthening the HCBS sector across the country.

One contributor to the unequal development between HCBS and institutional care, as well as the disparities between rural and urban areas and the Western and Eastern regions, is insufficient policy attention and distorted policy incentives aimed at meeting the specific bed supply targets set by the government [5]. To promote the nationwide development of HCBS, instead of concentrating on a few major cities, the central government initiated a 5-year pilot programme in 2016 that provided financial assistance to local authorities at selected pilot sites.

Previous studies in China showed that HCBS use was associated with better physical and mental health, a reduced likelihood of feeling lonely, and increased social participation among the Chinese older adults [14–19]. A recent study further assessed the impact of receiving HCBS on healthy ageing and provided policy implications for its development [20]. However, the effects of the 2016–2020 pilot programme that built capacity of HCBS nationwide on LTC needs remain unknown. Moving into the 14^th^ Five-Year Plan era (2021–2025), the central government is committed to further improving the supply of HCBS, building upon the achievement of the 2016–2020 pilot reform [21]. Evaluating the impact of the programme on the LTC needs of older adults is crucial at this stage, as it can provide valuable empirical evidence for future policymaking. In our study, we differentiated between the treated groups of cities covered by this pilot programme and the control group and evaluated the programme’s impact on the progression of care needs and the degree of unmet care needs, which were not covered in Su et al.’s study [20].

Using panel data from the China Health and Retirement Longitudinal Study (CHARLS), this study makes two contributions to the existing literature. To the best of our knowledge, this is the first study to evaluate the impact of this nationwide pilot HCBS programme on the number of unmet LTC needs among older adults with functional limitations and levels of LTC needs among older people in China. We distinguished treated and control cities based on government announcements and controlled for a set of city-by-year covariates that mirrored the selection criteria of the pilot sites in addition to individual-level covariates. We combined propensity score matching (PSM) with difference-in-differences (DiD) analysis to evaluate the programme’s impact on the covered population. Second, we further analysed the heterogeneous effects across key subgroups to assess whether the implementation of the pilot HCBS programme alleviated or exacerbated inequalities in LTC needs among older Chinese people.

### Institutional background

**Fig 1:**
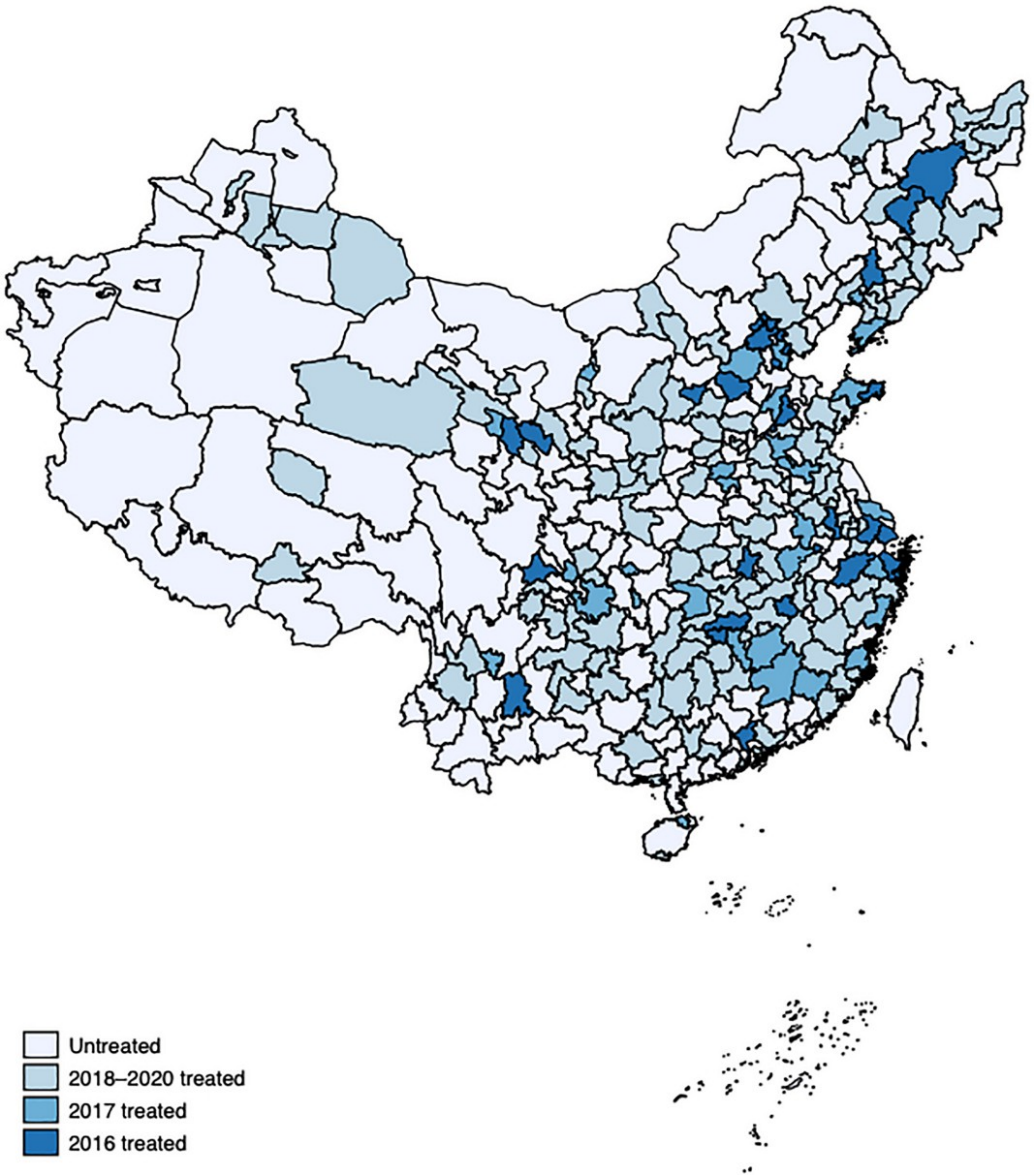
Pilot cities during 2016–2020. *Note*: The raw data used to create the map is publicly available from the National Platform for Common GeoSpatial Information Services (https://www.tianditu.gov.cn). The authors drew the map using the Stata software package (Release 16) and labelled the treated and untreated areas.

HCBS in China encounters a variety of common challenges, including a lack of qualified care workers, limited service options, low quality of care, lack of integration between health and social care, absence of stable profit models, inadequate incentive policies and legislation to encourage private investors’ involvement, and insufficient availability of HCBS [5, 22]. To address these challenges, in 2016, the central government decided to select areas for pilot reforms in the HCBS and provided financial assistance to local authorities for their initiatives to improve the HCBS [23]. From 2016 to 2020, the central government selected five cohorts consisting of 203 pilot sites (Fig 1), including prefectural cities or municipal districts, to enhance the capacity of the HCBS sector [24]. The total earmarked funding for this programme was 5 billion yuan, with an annual allocation of 1 billion yuan over the course of 5 years [24]. The objective of this programme was to identify successful HCBS practices through the provision of comprehensive services and active participation from social forces, which could then be adopted by other areas [23].

The selection process consisted of two stages [23]. The central government issued a call-for-application announcement, followed by the provincial Departments of Civil Affairs and Finance collecting and reviewing the application documents. Each province or municipality could recommend one or two prefectural cities or municipal districts for further consideration by the central government. Subsequently, the Ministry of Civil Affairs and Ministry of Finance convened an expert committee to evaluate the applicant sites based on the pilot criteria.

As stated in their announcement, the central government sought applications in which the local authorities of the applicant sites demonstrated a strong commitment to developing HCBS, possessed a solid foundation for this work, and had the desire and capability to carry out the pilot programme. Additionally, pilot regions were expected to represent a diverse range of population sizes, levels of population ageing, economic and social development, and local fiscal capacities.

The pilot funds primarily focus on fostering an ageing-friendly environment and building capabilities to develop HCBS for older people, with facile construction considered secondary [25]. The central government specified seven key support areas: (1) supporting the management and operation of HCBS facilities by social forces or the private sector through purchasing services, public-private partnerships, joint equity, and other approaches; (2) supporting nursing homes and other LTC institutions in extending their services to directly provide HCBS or offer technical support for HCBS facilities; (3) supporting the application of digital techniques in HCBS and facilitating the connection between the supply side and the demand side; (4) supporting the development of a professional LTC workforce in HCBS settings, strengthening training, developing incentives to attract and attain more professionals in the industry, and improving the quality of care; (5) promoting the standardisation of HCBS and developing third-party supervisory agencies and organisations through purchasing services; (6) supporting the active promotion of the integration of health care and LTC sectors; (7) supporting the development of HCBS in less developed urban or rural areas, utilising existing resources such as public nursing homes to build community-based care facilities to meet the LTC needs of those who are empty-nest, left-behind, disabled, solitary, and of advanced age. S1 Table in the Supplementary Material summarises typical community-based care facilities in China.

## Materials and methods

### Data and sample

We used data from the four waves of the CHARLS, a nationally representative panel survey. The baseline survey was conducted in 2011 (response rate: 80.5%) using multistage probability sampling to select 17,500 individuals aged ≥ 45 years from 150 counties across China. Follow-up surveys were conducted in 2013, 2015, and 2018. The latest CHARLS wave was conducted from July to December 2020, after COVID-19 began in China. To avoid plausible confounding effects of COVID-19 on the policy impacts related to LTC outcomes, we excluded 2020 from our observation period. This study did not require additional ethical approval because we obtained a public dataset after signing a Data Use Agreement with its holder. The CHARLS protocol was approved by the Ethical Review Committee of Peking University, China [26].

**Fig 2:**
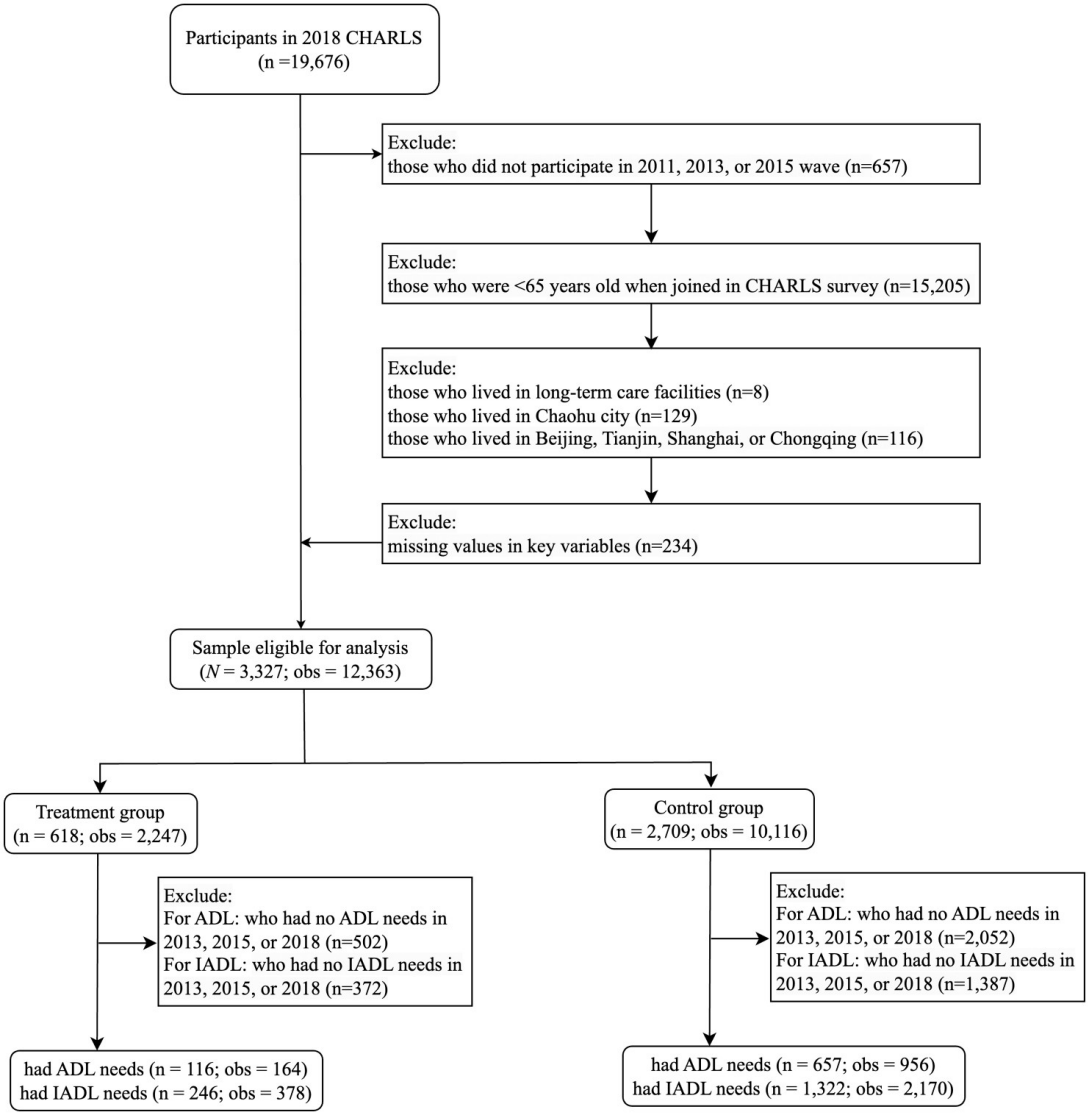
Sample selection. *Notes*: CHARLS, China Health and Retirement Longitudinal Study; ADL, activities of daily living; IADL, instrumental activities of daily living. Beijing, Tianjin, Shanghai, and Chongqing were excluded from the treated group in the main analysis because the pilot programme was only implemented in certain areas, which could not be identified in the CHARLS data. However, they were included in a robustness check of the main analysis results. Chaohu City was downgraded from a prefectural city to a county-level city in 2011; therefore, it was excluded from the analysis.

In this study, 19,676 respondents participated in the CHARLS 2018 wave. After excluding individuals who did not participate in any of the first three waves; those who were younger than 65 years of age when joined the CHARLS survey; those who were not community dwellers; those who lived in Beijing, Tianjin, Shanghai, Chongqing, or Chaohu cities; and those who had missing values in key variables, the final sample for baseline analysis had 3,327 older people, with 12,363 observations across the four waves. We excluded Beijing, Tianjin, Shanghai, and Chongqing from the treated group in the main analysis because the pilot programme was only implemented in certain parts of these cities, which could not be identified in the CHARLS data. However, they were included in a robustness check of the main analysis results. Chaohu was excluded from analysis because it was downgraded from a prefectural city to a county-level city in 2011. The study sample was divided into the treatment (*n* = 618; 2,247 observations) and control groups (*n* = 2,709; 10,116 observations). The treatment group includes individuals from the pilot cities that implemented the programme in 2016 or 2017, while the control group comprises individuals from cities that did not implement the programme during those years. Fig 2 illustrates the sample selection process. S2 Table in the Supplementary Material shows the distribution of sample size across the 2011–2018 waves in the CHARLS.

### Variable

#### Dependent variable

This study considered four dependent variables regarding LTC needs as follows: the number of unmet needs for assistance with activities of daily living (ADL), the number of unmet needs for assistance with instrumental activities of daily living (IADL), levels of ADL needs, and levels of IADL needs. The LTC needs were measured based on functional limitations in performing ADL and IADL tasks. The CHARLS asked participants to report whether they had difficulties performing five ADLs (dressing, bathing, eating, transferring, and toileting) and five IADLs (doing chores, cooking, taking medication, grocery shopping, and managing money). To ensure that these questions were related to LTC needs, the survey emphasised that participants should respond only if their limitations in ADL and IADL tasks were due to health or memory problems.

For each task, the respondents were given four options: ‘I have no difficulty’, ‘I have difficulty but can still do it’, ‘I have difficulty and need help’, and ‘I cannot do it’. The latter two options indicated a functional limitation in performing the task, we created 10 dichotomous variables for each task (1 = having a functional limitation with reference to performing the task; 0 = having no functional limitation to perform the tasks). Subsequently, we summed the number of difficulties reported by the participants in performing the five ADL and five IADL tasks and created a variable to measure the levels of ADL needs (range, 0–5; Cronbach’s alpha, 0.84) and a variable for the levels of IADL needs (range, 0–5; Cronbach’s alpha, 0.85). A higher score indicates a higher level of care need. Our approach of measuring care needs follows a previous study that indicates a single latent dimension underlying the functional abilities to perform ADL and IADL tasks [27]. This suggests that a simple count of ADLs and IADLs is a useful approximation of the severity of care needs [27].

Additionally, we created two variables to indicate the number of unmet needs for assistance with ADL and IADL. In the CHARLS 2013, 2015, and 2018 surveys, participants were asked whether they had received help with each functional task. We counted the number of unmet ADL needs among older adults with ADL limitations, and the same was performed for the number of unmet IADL needs. In the 2011 survey, unmet LTC needs were assessed in a general sense, rather than through specific tasks. Therefore, for the two variables regarding unmet care needs, we used only information from the 2013–2018 surveys.

#### Covariate

This study considered both individual and city characteristics that may be associated with LTC needs and the likelihood of living in a pilot city. To control for individual characteristics, we considered age (in years), sex (1 = female, 0 = male), marital status (1 = married, 0 = never married, widowed, separated, or divorced), urban-rural residence (1 = urban China, 0 = rural China), educational level (1 = illiterate, 2 = did not finish primary education, 3 = finished primary education, 4 = junior high school, 5 = high school and above), whether the respondent lived alone (1 = lived alone, 0 = co-resident with others), and type of social medical insurance (1 = Urban Employee Basic Medical Insurance, 2 = Urban Resident Basic Insurance, 3 = New Rural Cooperative Medical Scheme; 4 = Urban and Rural Resident Medical Insurance, 5 = Government Medical Insurance [*Gong Fei*], 6 = Uninsured), number of self-reported chronic diseases, and number of living children. Furthermore, we controlled for household spending as a proxy for household income. Household spending may reflect the standard of living more accurately than household income and can be applied to all household members, including women who may not have an income source or who are not the primary earners in the family [28]. The self-reported amount was divided by the square root of the number of household members to adjust for household size [29]. After adjusting for household size, a binary variable of ‘low income’ was created by assigning a score of 1 to respondents whose household spending fell into the lowest quartile, and a score of 0 was applied to everyone else.

Our study also controlled for a set of city-by-year covariates using statistics from the Statistical Yearbooks of China and each province, as well as demographic information from the Sixth National Census in 2010, the Seventh National Census in 2020, and the 2015 National 1% Population Sample Survey. We identified a set of control variables to capture the selection criteria for the pilot city using the best publicly available data in accordance with policy documents. These criteria require pilot areas to be representative of population size, population ageing, socioeconomic development, and local fiscal resources. At the prefectural city level, we identified five time-varying covariates: the number of counties, log-transformed total population (in ten thousand), log-transformed per capita GDP (in yuan), log-transformed general budget revenue (in ten thousand yuan), and share of the older population aged 65 years and above in the total population. Due to the lack of data on the share of the older population in the total population for 2013 in statistical yearbooks, we used the average of the 2010 and 2015 levels from the National Census and Population Sample Survey as a proxy for the 2013 data in our analysis.

#### Care utilisation variables

For the mechanism analysis, we created three variables regarding care utilisation. The 2018 CHARLS survey included an item on the self-reported utilisation of various types of HCBS in the past, including daycare centres, nursing homes, senior dining tables, or other facilities; regular physical examination; onsite visits; family beds; community nursing; health management; entertainment; and other services. Because this question was only introduced in the 2018 survey, we created two additional variables to indicate whether HCBS and/or informal care was received using information from the 2011 to 2018 surveys. The CHARLS includes an item on the sources of assistance in ADL and IADL tasks when the respondent reports any functional limitations. We created dichotomous variables to indicate whether an individual with any functional limitation reported receiving assistance from family members (1 = received informal care; 0 = did not receive informal care) and paid helpers, employees of facilities, nursing homes, or the community (1 = used HCBS; 0 = did not use HCBS).

### Empirical strategy

To identify the treatment and control groups, we obtained lists of pilot sites from government announcements. Using information from CHARLS, we identified 14 pilot cities that implemented the programme in 2016 and 8 pilot cities in 2017 as the treated group. It should be noted that the CHARLS does not cover all cities across the country, excluding some pilot sites. We excluded Beijing, Tianjin, Shanghai, and Chongqing from the treated group in the main analysis because the pilot programme was only implemented in certain parts of these cities and the CHARLS data did not allow us to identify individuals living in those specific parts. However, we included them in a robustness check of our main analysis results. Other cities that joined the programme between 2018 and 2020 were considered as the control group in our baseline analysis.

To evaluate the impact of the HCBS pilot programme on the number of unmet needs for assistance with ADL and IADL among older adults with functional limitations, as well as the levels of ADL and IADL needs among older Chinese people, we employed a conventional 2×2 DiD design. This DiD approach allows us to construct a counterfactual to estimate what would have happened to the outcomes if the HCBS pilot had not been introduced. The inclusion of pre-intervention data is crucial, as it accounts for any pre-existing differences between the groups, ensuring that post-intervention differences can be more confidently attributed to the intervention itself rather than to pre-existing trends. The treated group comprises cities that launched the pilot programme in 2016 or 2017, excluding four municipalities. The control group comprised those who were not covered by the programme in 2016 or 2017. If an individual lived in a city belonging to the treated group, *treat*_*ic*_ = 1; otherwise, *treat*_*ic*_ = 0. If the observation was measured in 2018, *post*_*t*_ = 1; otherwise, *post*_*t*_ = 0. Pre-treatment outcomes regarding care needs level were measured in 2011, 2013, and/or 2015, whereas pre-treatment outcomes regarding unmet care needs were measured in 2013 and/or 2015. Post-treatment outcomes were measured in 2018. The baseline DiD regression model for each outcome is as follows:

$$Y_{ict}=\beta_{0}+\beta_{1}HCBS_{ict}+\beta_{2}X_{ict}+\beta_{3}W_{ct}+\theta_{c}+\gamma_{t}+\varepsilon_{cit}$$ (1)

where *Y*_*ict*_ denotes the outcome variables of individual *i* living in city *c* in year *t*, including the number of unmet needs for assistance with ADL and IADL, as well as the levels of ADL and IADL needs. The *HCBS*_*ict*_ is an indicator of whether the city introduced the pilot programme in 2016 or 2017, which represents *treat*_*ic*_ × *post*_*t*_. *X*_*ict*_ is a vector of individual covariates and *W*_*ct*_ is a set of city-by-year covariates. *θ*_*c*_ is city fixed effect; *γ*_*t*_ is year fixed effect. *treat*_*ic*_ and *post*_*t*_ are omitted from the model because they are taken into account by city and time fixed effects. *ε*_*cit*_ is the error term. *β*_0_, *β*_1_, *β*_2_, and *β*_3_ are the parameters to be estimated; *c* represents city; *i* represents individual; *t* represents year. Standard errors are clustered at the city level to account for possible correlations in outcomes between older adults in the same city.

One concern is that the implementation of this programme is not random. In addition to controlling for a set of city-by-year covariates to account for the selection criteria of the pilot cities, we controlled for city fixed effects to further account for unobservable time-invariant heterogeneities across cities.

Another concern is potential selection bias related to certain observable characteristics, such as the sole living arrangement. To enhance the similarity of observables between the treated and control groups, we combined DiD regression with the PSM method to achieve ‘double robustness’, following the approach of Imbens and Wooldridge [30]. First, we estimated a logit model for the probability of being in the treated group based on a set of observable characteristics in each wave, including age, sex, educational level, urban residence, marital status, household income level, living alone, medical insurance type, number of chronic diseases, and number of living children. As we have an imbalanced panel of cities and individuals observed over the four waves, matching of individuals was implemented on a year-by-year basis using the aforementioned covariates [31, 32]. We used two-nearest neighbour matching without a replacement method. We then conducted DiD analyses with the matched sample within common support, ensuring that the two groups had a similar probability of being in the treated group. Observations outside common support were excluded from the analyses. Following the matching process, all observable characteristics were well-balanced, and the distributions of the propensity scores were more similar between the treated and control groups (S1 Fig and S3 Table in the Supplementary Material).

## Results

### Descriptive statistics

Table 1 presents summary statistics for the full sample, as well as the treatment and control groups during the pre-treatment and post-treatment periods. The *t*-test indicated the significance level of the pairwise comparisons before the implementation of the HCBS programme. On average, older individuals had 0.2 functional limitations in ADL and 0.55 limitations in IADL. Among those with ADL limitation, the average number of unmet needs for assistance with ADL was 0.86, whereas among those with IADL limitation, the average number of unmet needs for assistance with IADL was 0.4. During the pre-treatment period (2011–2015), the treated group had lower levels of ADL and IADL needs than the control group.

**Table 1:** Sample characteristics (without matching).

Variable	Full sample	Pre-intervention		Post-intervention		*t-test*
		Treated	Control	Treated	Control	
Level of ADL needs (0–5)	0.20	0.11	0.15	0.28	0.38	2.75**
	(0.73)	(0.52)	(0.59)	(0.87)	(1.04)	
Level of IADL needs (0–5)	0.55	0.33	0.45	0.73	0.94	4.63**
	(1.17)	(0.88)	(1.02)	(1.38)	(1.53)	
Age (65–108)	74.38	73.20	73.03	78.07	77.92	
	(5.89)	(5.54)	(5.53)	(5.36)	(5.35)	
Female (%)	51.3	52.4	51.1	52.6	50.8	
Marital status (%)	69.7	74.0	72.1	64.1	61.8	
Educational attainment (%)						
Illiterate	40.1	33.5	41.7	33.2	41.5	
Did not finish primary education	18.9	20.2	18.8	19.6	18.8	
Finished primary education	23.7	22.8	23.9	23.0	23.8	
Junior high school	10.1	10.8	9.8	11.3	9.9	
High school and above	7.1	12.7	5.8	12.9	6.0	
Social medical insurance (%)						
Urban Employee Basic Medical Insurance	13.0	21.7	9.9	24.1	13.6	
Urban Resident Basic Medical Insurance	6.2	4.6	3.9	7.6	12.8	
New Rural Cooperative Medical Scheme	52.2	57.4	73.0	4.7	3.3	
Urban and Rural Resident Medical Insurance	17.5	2.8	1.3	55.8	61.9	
Government Medical Insurance	3.3	5.3	3.2	3.7	2.3	
Uninsured	7.8	8.2	8.7	4.1	6.1	
Urban residence (%)	37.3	53.9	33.1	56.5	34.2	
Low-income level (%)	25.3	21.8	25.8	20.6	27.3	
Living alone (%)	17.6	14.9	15.3	22.7	24.2	
Agricultural *hukou* (%)	76.5	64.9	79.4	63.4	78.5	
Number of chronic diseases	0.63	0.58	0.56	0.76	0.84	
	(1.16)	(1.17)	(1.16)	(1.03)	(1.14)	
Number of surviving children	3.72	3.41	3.71	3.61	3.97	
	(1.72)	(1.76)	(1.75)	(1.62)	(1.62)	
Number of counties	9.73	12.00	9.24	11.89	9.23	
	(4.11)	(4.64)	(3.82)	(4.64)	(3.80)	
Log (population size in 10,000)	6.19	6.48	6.12	6.54	6.13	
	(0.50)	(0.41)	(0.49)	(0.43)	(0.49)	
Log (per capital GDP in yuan)	10.52	11.01	10.30	11.34	10.63	
	(0.58)	(0.55)	(0.50)	(0.49)	(0.45)	
Log (budgeted revenue in 10,000 yuan)	14.03	15.16	13.66	15.57	14.03	
	(0.96)	(0.78)	(0.74)	(0.75)	(0.72)	
Share of older population	10.99	9.68	9.76	12.89	14.73	
	(3.09)	(1.96)	(1.88)	(3.11)	(3.08)	
East China (%)	30.8	46.6	27.5	45.8	27.1	
*Observations*	*12*,*363*	*1*,*629*	*7*,*407*	*618*	*2*,*709*	
Number of unmet ADL needs (0–4)	0.86	0.84	0.89	0.79	0.84	0.40
	(1.09)	(1.01)	(1.09)	(1.14)	(1.10)	
*Observations*	*1*,*120*	*75*	*495*	*89*	*461*	
Number of unmet IADL needs (0–4)	0.40	0.52	0.44	0.25	0.37	–1.31
	(0.74)	(0.87)	(0.74)	(0.64)	(0.72)	
*Observations*	*2*,*548*	*204*	*1*,*199*	*174*	*971*	

*Notes*: For care needs level, pre-intervention considered observations from the 2011, 2013, and 2015 waves; post-intervention considered observations from the 2018 wave. For the numbers of unmet needs for assistance with ADL and IADL, pre-intervention only considered observations from the 2013 and 2015 waves. Standard deviations are shown in parentheses. A two-sample *t*-test was used for pairwise comparisons of the treated and control groups during the pre-intervention period, assuming unequal variance. GDP, gross domestic product; ADL, activities of daily living; IADL, instrumental activities of daily living. A significance level of 5% was denoted by *.

### Main effects of the programme

Table 2 shows the estimated effects of the HCBS programme on the number of unmet needs for assistance with ADL and IADL, as well as the levels of ADL and IADL needs. Column 1 shows that under DiD without matching specifications, the implementation of the HCBS programme reduced the number of unmet needs for IADL by 20.9 percentage points and slowed IADL functional decline by 9.9 percentage points. These results were statistically significant. However, we observed no significant effect on the number of unmet needs for ADL or level of ADL needs. In Column 2, based on the PSM-matched sample, the estimates experienced minimal changes in magnitude, while remaining consistent with the results in Column 1.

**Table 2:** Programme impact on the number of unmet care needs and the levels of long-term care needs.

Dependent variable	DiD	DiD with matching
	(1)	(2)
Number of unmet ADL needs	–0.054	–0.057
	(0.202)	(0.204)
R-squared	0.1688	0.1681
Observations	1,120	1,117
Number of unmet IADL needs	–0.209*	–0.213*
	(0.104)	(0.105)
R-squared	0.1114	0.1118
Observations	2,548	2,546
Levels of ADL needs	–0.051	–0.050
	(0.040)	(0.041)
R-squared	0.0769	0.0767
Observations	12,363	12,319
Levels of IADL needs	–0.099*	–0.097*
	(0.047)	(0.048)
R-squared	0.1584	0.1581
Observations	12,363	12,319

*Notes*: Robust standard errors clustered at the city level are reported in parentheses. All regressions control for city fixed effects, year fixed effects, individual covariates (including age, sex, marital status, educational level, urban residence, low-income level, living alone, type of social medical insurance, number of chronic diseases, and number of living children), and city covariates (including the number of counties, logarithm of population size, logarithm of per capita gross domestic product, logarithm of public budgeted revenue, and proportion of older people in the total population). ADL, activities of daily living; IADL, instrumental activities of daily living. A significance level of 5% was denoted by *.

To examine the potential differential impacts between the 2016 and 2017 pilot cities, we constructed a variable indicating whether the treated city introduced the programme in 2016 or 2017. This variable, along with its interaction term with the *HCBS* variable, were added into the model. The lack of significance in the interaction term suggests no difference in programme effects between the pilot cities in 2016 and 2017 (not reported).

### Heterogeneity

Heterogeneity analyses were conducted to examine whether the implementation of the HCBS programme was beneficial to people across different subgroups. The pace of social and economic development is often highly unequal across different regions and between rural and urban areas. Therefore, we investigated the impacts of the programme according to geographical area (Eastern China vs. other regions) and urban-rural residence (urban vs. rural). Considering that the HCBS programme is targeted at the oldest old and solitary residents, it is important to evaluate the impacts of the programme by age group (age 80 and above vs. age 65–79) and living arrangement (living alone vs. co-resident). We also conducted heterogeneity analyses by household low-income status (low-income vs. not low-income) and *hukou* type (agricultural vs. non-agricultural *hukou*) because they have been identified as two significant risk factors for unmet LTC needs among Chinese older adults [33, 34].

**Fig 3:**
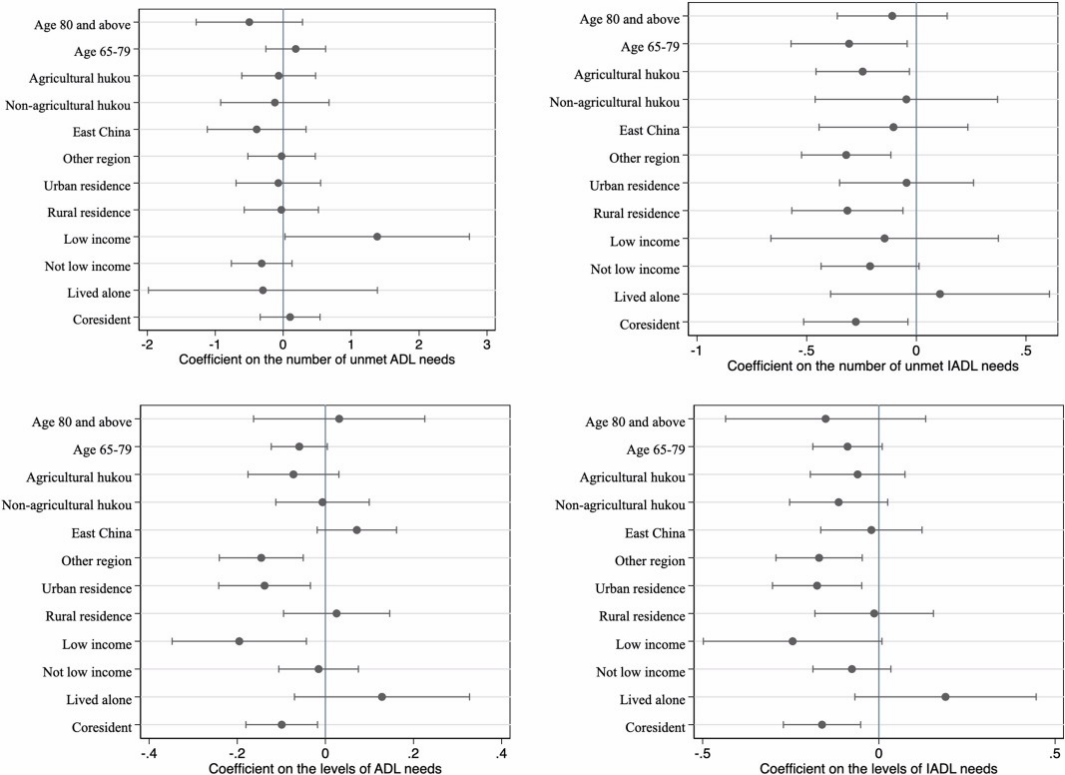
Heterogeneity analysis with subgroups (DiD with matching). *Notes*: The figure shows the DiD estimates with the PSM-matched sample for the effect of the HCBS programme on the respective outcomes by age group, *hukou* type, geographical area, urban-rural residence, household low-income status, and living arrangement. We divided the sample into subgroups and conducted regression analysis. The dots mark the point estimates, and the lines indicate 95% confidence intervals. All regressions controlled for year fixed effects, city fixed effects, individual-level covariates, and city-by-year covariates.

We created interaction terms between the *HCBS* variables and subgroup indicators to measure heterogeneity across each subgroup. Table 3 shows the results of the heterogeneous analysis with the interaction term using the PSM-matched sample. The specifications in Table 3 are identical to those in Table 2 except for the inclusion of an interaction term and a subgroup indicator. In addition, we divided the PSM-matched sample into subgroups, as shown in Fig 3. We considered only the subgroups that showed group differences in Fig 3 and significant interaction terms in Table 3, indicating heterogeneous programme impacts.

**Table 3:** Heterogenous analysis with interaction term (DiD with matching).

	Number of unmet ADL needs	Number of unmet IADL needs	Levels of ADL needs	Levels of IADL needs
	(1)	(2)	(3)	(4)
*Panel A*. *By the oldest old (>80 years old) or younger*				
Policy × oldest	–0.132	0.201	0.153*	0.236
	(0.296)	(0.122)	(0.070)	(0.122)
Policy	0.018	–0.317*	–0.107**	–0.185**
	(0.212)	(0.125)	(0.035)	(0.055)
*Panel B*. *By non-agricultural/agricultural hukou*				
Policy × agricultural *hukou*	0.146	–0.054	–0.039	0.170*
	(0.297)	(0.091)	(0.067)	(0.080)
Policy	–0.175	–0.173	–0.026	–0.204**
	(0.260)	(0.143)	(0.049)	(0.056)
*Panel C*. *By East China or other regions*				
Policy × East	0.172	0.263	0.141**	0.130*
	(0.299)	(0.186)	(0.049)	(0.060)
Policy	–0.135	–0.331***	–0.112**	–0.154**
	(0.228)	(0.100)	(0.043)	(0.051)
*Panel D*. *By Urban/rural residence*				
Policy × urban residence	0.074	0.143	–0.035	–0.165
	(0.249)	(0.078)	(0.075)	(0.099)
Policy	–0.093	–0.283**	–0.030	–0.005
	(0.237)	(0.099)	(0.067)	(0.087)
*Panel E*. *By household low-income status*				
Policy × low income	0.845	–0.021	–0.124	–0.050
	(0.577)	(0.156)	(0.066)	(0.119)
Policy	–0.158	–0.208*	–0.024	–0.086
	(0.194)	(0.105)	(0.047)	(0.054)
*Panel F*. *By living arrangement*				
Policy × living alone	–0.383	0.175	0.219**	0.213
	(0.248)	(0.167)	(0.079)	(0.130)
Policy	0.05	–0.253*	–0.100*	–0.145**
	(0.205)	(0.107)	(0.041)	(0.054)
Observations	1,117	2,546	12,319	12,319

*Notes*: Standard errors are in brackets. ADL, activities of daily living; IADL, instrumental activities of daily living. Significance levels of 5%, 1%, and 0.1% were denoted by *, **, and ***.

As shown in Table 3 and Fig 3, the implementation of the HCBS reduced the levels of ADL and IADL needs among individuals residing outside the Eastern region but did not show a significant reduction among those living in the Eastern region. It also decreased the level of ADL needs among individuals living with others but did not show a significant decrease among those living alone. The results obtained using the unmatched sample, presented in S4 Table and S2 Fig in the Supplementary Material, were consistent with the baseline analysis.

### Mechanism

We investigated the mechanism by which the programme delayed the increase in levels of IADL needs over time. Using information from the 2011 to 2018 CHARLS surveys regarding the sources of assistance in ADL and IADL tasks when respondents reported any functional limitations and employing the same specification as in Eq (1), we found that the introduction of the HCBS programme significantly increased the use of HCBS by 1.5 percentage points but had no impact on the receipt of informal care (not reported). Although the use of the HCBS variable appears to employ a narrow definition as it is restricted to those already displaying functional limitations, our findings on the positive effects of the programme in decelerating the rising level of IADL needs may be explained by the improved availability and subsequent utilisation of HCBS.

### Robustness checks

**Fig 4:**
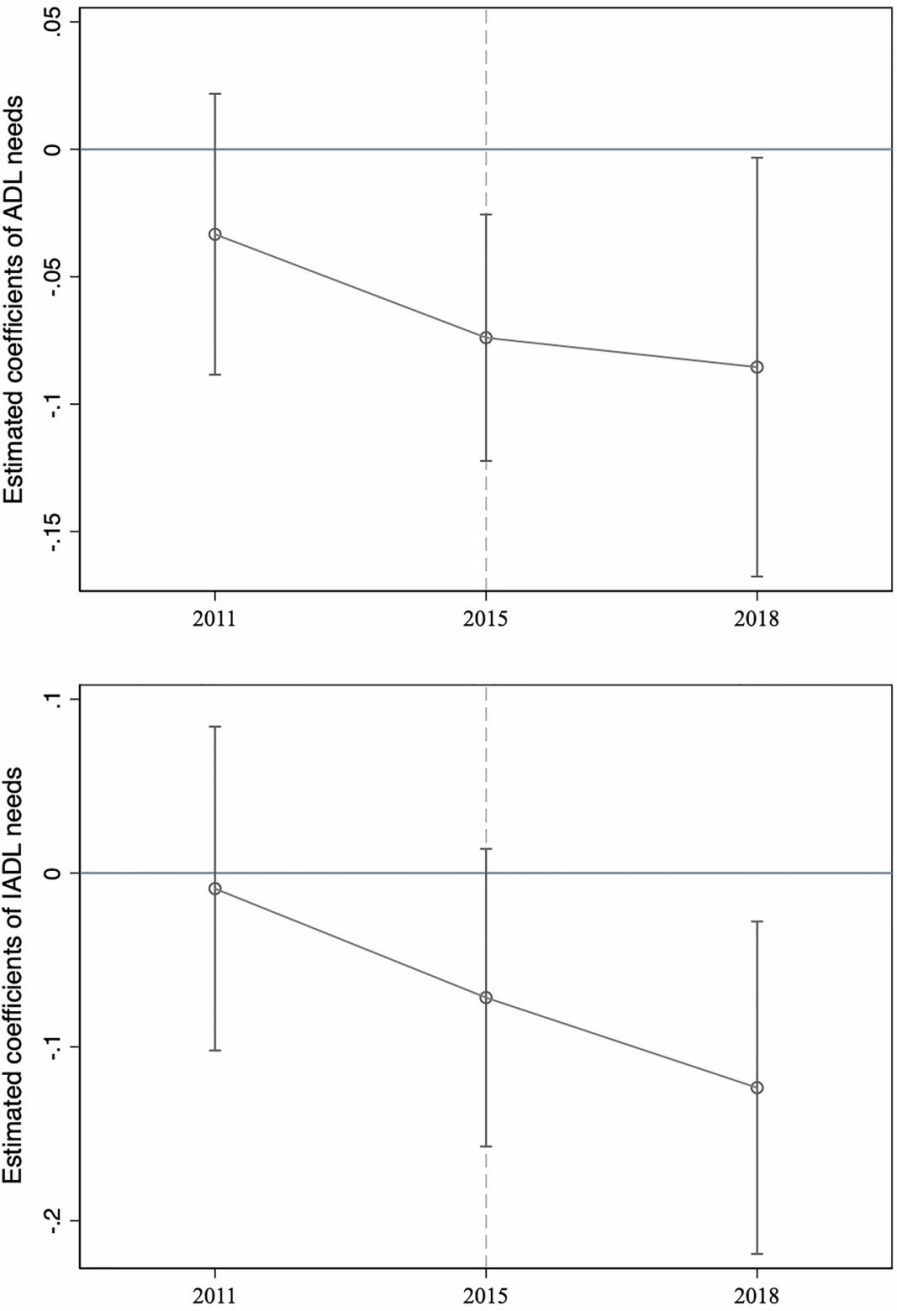
Parallel trend checks (PSM-matched sample). *Notes*: This figure depicts the coefficients and 95% confidence intervals of event studies for the programme’s effects on the levels of ADL and IADL needs. The year 2013 was excluded as the reference year. All regressions controlled for year fixed effects, city fixed effects, individual-level covariates, and city-by-year covariates.

The key assumption of the DiD specification is that the selection of pilot cities is unrelated to other factors that influence the changes in each outcome. To examine this assumption, we conducted a common trend test to assess whether the treated and control groups exhibited similar time trends for each outcome before the programme implementation. Following previous study’s approach [35], we conduct event study analyses to evaluate the effects on each outcome. Using the 2011–2018 panel with PSM, we depict the coefficients from an alternative specification of Eq (1), in which the interaction term *HCBS*_*ict*_ is replaced with a sequence of timing dummies indicating the survey years before and after the implementation of the HCBS programme. In Fig 4, the X-axis represents each survey year, with the event time of 2015, which was the year closest to the actual reform year of 2016 or 2017. The reference category was the 2013 wave, which was excluded from the benchmark category. The estimate for the pre-reform period from 2011 to 2015 was not significant for the levels of IADL needs, indicating that the pre-reform trends of this outcome in the treated group were not different from those in the control group. From 2011 to 2013, the levels of ADL needs between the treated and control groups showed similar trends. Using the original sample without matching, common trend tests yielded consistent findings (see S3 Fig in the Supplementary Material).

For robustness checks, we considered the LTC insurance pilot policy that was implemented during the implementation period of the HCBS programme, which could have influenced the functional abilities of older adults and their utilisation of LTC services. To address potential unobserved confounding effects, we created a dummy variable for the policy and controlled for its fixed effects. Additionally, we added a community fixed effect to control for unobservable time-invariant characteristics. We also applied alternative definitions for the treated and control groups, which involved excluding the 2018 pilot cities from the control group and including Beijing, Tianjin, Shanghai, and Chongqing cities in the treated group.

Table 4 presents the results of these robustness checks with the PSM-matched samples. The results indicate that the fixed effects of the LTC insurance policy were already being accounted for by city fixed effects. The insignificant programme impact on the number of unmet ADL needs and the levels of ADL needs remained unchanged. However, some discrepancies were observed regarding the number of unmet IADL needs. Adjusting for community fixed effects or excluding the 2018 pilot cities from the control group yielded marginally insignificant results at the 10% significance level. For the levels of IADL needs, including the four cities in the treated groups yielded marginally insignificant results at the 10% significance level, whereas excluding 2018 from the control group led to insignificant results. Robustness checks using the original sample without matching produced similar results in terms of significance level and magnitude (see S5 Table in the Supplementary Material).

**Table 4:** Robustness checks for programme impact (DiD with matching).

	Number of unmet ADL needs		Number of unmet IADL needs		Levels of ADL needs		Levels of IADL needs	
	(1)	(2)	(1)	(2)	(1)	(2)	(1)	(2)
Policy	–0.057	–0.041	–0.213*	–0.221	–0.050	–0.053	–0.097*	–0.098*
	(0.204)	(0.234)	(0.105)	(0.116)	(0.041)	(0.041)	(0.048)	(0.049)
LTCI fixed effect	YES		YES		YES		YES	
Community fixed effect		YES		YES		YES		YES
Observations	1,117	1,042	2,546	2,510	12,319	12,319	12,319	12,319
R-squared	0.1681	0.2999	0.1118	0.1949	0.0767	0.1201	0.1581	0.2011
	Number of unmet ADL needs		Number of unmet IADL needs		Levels of ADL needs		Levels of IADL needs	
	(3)	(4)	(3)	(4)	(3)	(4)	(3)	(4)
Policy	–0.001	–0.057	–0.189	–0.213*	–0.039	–0.037	–0.080	–0.106
	(0.210)	(0.204)	(0.106)	(0.105)	(0.042)	(0.038)	(0.050)	(0.057)
Observations	1,016	1,117	2,352	2,546	11,270	12,752	11,270	12,752
R-squared	0.1794	0.1681	0.1092	0.1118	0.0789	0.0781	0.1569	0.1591

*Notes*: Robust standard errors clustered at the city level are reported in parentheses. All regressions control for city fixed effects, year fixed effects, individual covariates (including age, sex, marital status, educational level, urban residence, low-income level, living alone, type of social medical insurance, number of chronic diseases, and number of living children), and city covariates (including the number of counties, logarithm of population size, logarithm of per capita gross domestic product, logarithm of public budgeted revenue, and proportion of older people in the total population). In addition, Model (1) considers the fixed effects of the long-term care insurance pilot policy. Model (2) considered the community fixed effect and did not adjust for urban residences because of collinearity; some communities were omitted because of collinearity. Model (3) excludes the 2018 pilot cities from the control group. Model (4) includes Beijing, Tianjin, Shanghai, and Chongqing in the treated group. ADL, activities of daily living; IADL, instrumental activities of daily living; LTCI, long-term care insurance. A significance level of 5% was denoted by *.

## Discussion

In response to the rapidly expanding needs for HCBS among older adults in China, the central leadership implemented an HCBS programme from 2016 to 2020 to strengthen HCBS at pilot sites. Using nationally representative longitudinal data, we assessed the impact of the programme on the numbers of unmet needs for assistance with ADL and IADL as well as the levels of ADL and IADL needs. Additionally, we explored the heterogeneous impact of the programme across key subgroups. The key findings are summarised and discussed as follows.

First, our study provides evidence that the HCBS programme effectively reduced the number of unmet needs for assistance with IADL among older adults with IADL limitations. However, it did not have a significant impact on ADL needs. To strengthen their HCBS, local authorities at pilot sites implemented a variety of measures, such as enhancing the availability of community-based canteens for older people in rural areas, increasing the provision of bathing services, developing innovative institutional mechanisms for the government purchase of private HCBS services for local older residents, promoting the development of respite care for informal caregivers of older people living with functional disabilities or dementia, exploring the use of digital devices to facilitate the provision of HCBS, expanding professional services from nursing homes to older people’s homes, developing a professional workforce, and promoting ageing-friendly housing adaptations [24].

With these efforts, it is not surprising to observe the positive effect of implementing the HCBS programme in mitigating the unmet needs of older people. Indeed, our findings show that the programme significantly increased HCBS use among older adults with functional limitations. Although we found no differences in programme effects between the 2016 and 2017 pilot cohorts, the Average Treatment Effect on the Treated may have been underestimated because our study design assumed that all pilot cities began intervention in 2016, despite differences in programme exposure duration.

The differential effects on unmet needs for assistance with ADL and IADL may be attributed to several factors. Help with ADL tasks is typically more personal in nature than that with IADL tasks and thus may pose greater challenges in reaching out to older people with care needs. Chinese older adults may prefer to receive publicly funded services to meet their IADL care needs and family caregivers for assistance with ADL tasks. Many older adults tend to receive personal care, such as hygiene assistance, from familiar individuals, particularly family members. Recent evidence supports this preference for informal care provided by family members over formal care among long-term care insurance beneficiaries in China [36]. It seems that the HCBS pilot programme is more effective at meeting older people’s needs for IADL care. These findings have important policy implications. On the one hand, increasing the provision of HCBS should be able to play an important role in reducing the unmet IADL care needs in the Chinese older population. On the other hand, HCBS alone may not be enough in addressing the issue of unmet needs in the context of population ageing and rising care demand. Given the important role of family caregivers in meeting older people’s ADL care needs, stepping up support for family caregivers (e.g., respite services) so that they have the capacity and capability to provide care for their loved ones in the long run is equally important.

Second, we found that the implementation of the programme slowed the progression of IADL limitations among older adults in pilot areas but had no effect on ADL needs. The discrepancy in the effects may be attributed to the fact that ADL tasks typically require more physical abilities that are essential for independent living, such as bathing and eating, whereas IADL tasks tend to require more complex planning and organisational skills to live independently in the community, such as managing finances and medications. Another possibility is that IADL care needs represent a lower level of care needs than ADL care needs. It is easier to help people recover from their IADL care needs through interventions. Accordingly, improvements in HCBS could benefit older people in terms of IADL rather than ADL needs.

The increased accessibility and subsequent rise in the utilisation of HCBS in the pilot cities may explain the causal relationship between HCBS programme implementation and IADL needs. Indeed, our study found that the programme’s implementation increased HCBS use among older adults with functional limitations. The explanation that increased HCBS use can reduce IADL limitations is consistent with Verbrugge and Jette’s Disablement Process Model [7] and empirical evidence from a recent study of the Chinese older population [20]. Previous studies also showed that HCBS use was associated with better physical and mental health, a reduced likelihood of feeling lonely, and increased social participation among Chinese older adults [14–19]. It has been suggested that increased social participation and improved nutritional intake benefiting from the utilisation of HCBS are associated with a reduction in functional decline in IADL [37, 38].

Third, we uncovered heterogeneous effects of the programme across subgroups, with considerable inequality implications. The implementation of the HCBS programme tended to alleviate disparities in the levels of ADL and IADL needs among older adults living in the Eastern and other regions. However, this exacerbated the disparities in ADL needs between those who lived alone and coresidents. More policy attention should be paid to those living alone, thereby minimising existing disparities in functional limitations among older Chinese adults.

Our study provides valuable evidence for policymakers committed to improving HCBS and aged care services. During the 14^th^ Five-Year Plan period (2021–2025), the Chinese central government committed to further promoting HCBS. The Ministries of Civil Affairs and Finance launched successive rounds of the HCBS pilot programme from 2021 to 2023. This initiative aimed to provide subsidies for constructing home care beds or developing home care services for financially disadvantaged and functionally disabled older adults. The programme monitors indicators such as HCBS infrastructure, facility construction, service provision, and quality (process indicators), and the fiscal investment of local authorities. Our findings indicate the positive effects of the precursor pilot programme conducted between 2016 and 2020 in limiting unmet needs for assistance with IADL and delaying increases in IADL levels. Based on our findings of heterogeneous effects, future efforts should prioritise older adults who live alone. During the implementation of new pilot programmes, it is imperative to measure health outcome indicators that are not considered by the ongoing programme to conduct a comprehensive evaluation of the impacts of improved HCBS infrastructure and accessibility on the well-being of older beneficiaries. These efforts can also benefit the ongoing implementation of the Basic Aged Care Services List, a policy recently enacted by the central leadership in response to rapid population ageing, as some of the above-mentioned services are covered in the national list [39].

This study has several limitations. First, we could not fully address the self-selection of pilot sites. Local authorities voluntarily applied for grants and submitted supporting documents to demonstrate their commitment to improving their HCBS, which was a criterion for selection by the government-convened committee. Although we controlled for a set of city-by-year covariates and city fixed effects, we could not fully account for the endogeneity caused by the self-selection of the pilot cities. Second, we did not have access to exact selection criteria. However, based on government announcements, we used the best publicly available data to control for a set of city-level covariates, thereby addressing selection bias at the city level arising from the pilot city selection process. Third, as there were no observations in 2016 or 2017, when the programme was implemented, we could not determine the actual year of programme implementation using a parallel trend test. Instead, we assumed that 2015 was the reform year. Because the 2015 survey was conducted between July 2015 and January 2016, our assumption regarding 2015 as the reform year may pose a limited threat to the estimation. Moreover, owing to the absence of data in 2011, we could not conduct event studies on the two unmet needs outcomes. Fourth, it should be noted that the policy intervention is to provide financial assistance to support local authorities in implementing supply-side initiatives to enhance HCBS. The specific design of the supply-side intervention on HCBS exhibits significant variations across pilot sites. Therefore, our results should be interpreted as the impact of this programme that the central government selected and incentivised certain cities to improve the supply of HCBS in their administrative areas, rather than construing as the effects of any specific interventions at the practice level on LTC needs among older persons. Future studies may focus on data collection and analyses to investigate the effects of differential designs in supply-side HCBS interventions. Fifth, the CHARLS surveys used for analysis were conducted before the completion of the HCBS programme, with the last survey in 2018, two years before the programme’s conclusion in 2020. Therefore, the data may not fully capture the overall impact of the entire HCBS programme. Sixth, given that not all pilot cities are covered by CHARLS, the absence of data may introduce selection bias if the uncovered pilot cities systematically differ from the covered cities in ways that could affect the policy impacts on LTC needs. Although CHARLS is nationally representative, which helps mitigate this risk, we cannot fully rule out the possibility of potential selection bias. Seventh, as in many other studies [40, 41], the number of observations for unmet ADL may be small. The insignificant result regarding policy impacts on unmet ADL needs might be due to limited statistical power.

## Conclusion

Our study provides empirical evidence of the positive effects of implementing the HCBS pilot programme on slowing IADL functional decline among older adults and reducing the number of unmet needs for assistance with IADL among those with IADL limitations. We also observed heterogeneous effects, particularly among disadvantaged older adults living alone. These findings have significant implications for future policy initiatives aimed at strengthening the HCBS in China and alleviating potential inequalities in the functional limitations of older adults.

## Supporting information

S1 Table Typical home- and community-based care facilities for older adults in China. (DOCX)

S2 Table Distribution of sample size across 2011–2018 waves in CHARLS. (DOCX)

S3 Table Balance test of covariates (year-by-year matching). (DOCX)

S4 Table Heterogenous analysis with interaction term (DiD without matching). (DOCX)

S5 Table Robustness checks for programme impact (DiD without matching). (DOCX)

S1 Fig Distribution of propensity scores before and after matching. (DOCX)

S2 Fig Heterogeneity analysis with subgroups (DiD without matching). (DOCX)

S3 Fig Parallel trend checks (without matching). (DOCX)
