# Supplementary material for: Evaluating the impact of a pilot programme for home- and community-based services on long-term care needs among older adults in China
Source: PLoS One. 2024 Nov 21;19(11):e0311616. doi: 10.1371/journal.pone.0311616 (PMC11581224; doi:10.1371/journal.pone.0311616)
Supplement: S1 Table — (DOCX) [file pone.0311616.s001.docx]

**S1 Table. Typical home- and community-based care facilities for older adults in China**

| Facility | Services |
| --- | --- |
| Community day care centre  (*ri-jian-zhao-liao-zhong-xin*) | In urban areas, the centres provide meals, social activities, basic diagnostic health check-ups, personal care, rehabilitation through physiotherapy exercise and use of rehabilitation equipment, psychological comfort and companionship, education, medical service referral, and other services for older people who can live independently or those who can live independently with some assistance. |
| Happiness Home (*xing-fu-yuan*) | In rural areas, the Happiness Homes are a type of community-based care facilities where older people in the village support each other without employing service staff. |
| Community station (*tuo-lao-suo*) | In urban areas, community stations provide day care and temporary services, as well as respite services for family caregivers. |
| Social activities centre  (*lao-nian-huo-dong-zhan*) | In urban areas, the centres organise a variety of social activities for older people. |

Sources: Feng et al. (2020) and National Standards by the Ministry of Civil Affairs
