# Supplementary material for: Evaluating the impact of a pilot programme for home- and community-based services on long-term care needs among older adults in China
Source: PLoS One. 2024 Nov 21;19(11):e0311616. doi: 10.1371/journal.pone.0311616 (PMC11581224; doi:10.1371/journal.pone.0311616)
Supplement: S2 Table — (DOCX) [file pone.0311616.s002.docx]

**S2 Table. Distribution of sample size across 2011–2018 waves in CHARLS**

| 2011 | 2013 | 2015 | 2018 | n |
| --- | --- | --- | --- | --- |
| Yes | Yes | Yes | Yes | 2,613 |
| No | Yes | Yes | Yes | 383 |
| Yes | No | Yes | Yes | 127 |
| Yes | Yes | No | Yes | 99 |
| No | No | Yes | Yes | 48 |
| Yes | No | No | Yes | 29 |
| No | Yes | No | Yes | 28 |
