# Supplementary material for: Evaluating the impact of a pilot programme for home- and community-based services on long-term care needs among older adults in China
Source: PLoS One. 2024 Nov 21;19(11):e0311616. doi: 10.1371/journal.pone.0311616 (PMC11581224; doi:10.1371/journal.pone.0311616)
Supplement: S3 Table — (DOCX) [file pone.0311616.s003.docx]

**S3 Table. Balance test of covariates (year-by-year matching)**

| Before matching | (1) | (2) | (3) | (4) |  | After matching | (1) | (2) | (3) | (4) |
| --- | --- | --- | --- | --- | --- | --- | --- | --- | --- | --- |
|  | 2011 | 2013 | 2015 | 2018 |  |  | 2011 | 2013 | 2015 | 2018 |
| Age | 0.0134 | 0.0154 | 0.011 | 0.0178 |  | Age | -0.005 | 0.0284^*^ | 0.0222 | -0.0052 |
|  | (0.9512) | (1.2071) | (0.839) | (1.343) |  |  | (-0.2743) | (1.6558) | (1.3296) | (-0.3270) |
| Female | 0.1912^*^ | 0.2174^**^ | 0.1835^*^ | 0.2402^**^ |  | Female | -0.0878 | 0.1502 | -0.0027 | 0.0799 |
|  | (1.7213) | (1.976) | (1.771) | (2.3171) |  |  | (-0.5353) | (1.0131) | (-0.0182) | (0.5471) |
| Educational attainment | 0.1142 | 0.1134 | 0.0938 | 0.116 |  | Educational attainment | -0.0267 | 0.1235 | 0.0471 | 0.0355 |
|  | (1.3917) | (1.4249) | (1.1106) | (1.5287) |  |  | (-0.2938) | (1.4134) | (0.5229) | (0.4443) |
| Urban residence | 0.7198^**^ | 0.6074^*^ | 0.6456^**^ | 0.7013^**^ |  | Urban residence | 0.0396 | 0.3232 | 0.3137 | 0.134 |
|  | (2.2806) | (1.8945) | (1.995) | (2.069) |  |  | (0.1263) | (0.9851) | (0.9643) | (0.3741) |
| Marital status | 0.2402 | 0.2711 | 0.1703 | 0.1423 |  | Marital status | -0.1614 | -0.0624 | -0.2311 | -0.1103 |
|  | (1.401) | (1.5442) | (1.0925) | (0.931) |  |  | (-0.7282) | (-0.2440) | (-1.0041) | (-0.4952) |
| Low-income status | -0.0576 | -0.1167 | -0.1705 | -0.16 |  | Low-income status | 0.0133 | 0.2095 | 0.2291 | -0.0273 |
|  | (-0.2944) | (-0.6727) | (-1.0841) | (-1.1360) |  |  | (0.0567) | (1.0336) | (1.288) | (-0.1515) |
| Living alone | 0.1801 | 0.1875 | -0.0339 | -0.0277 |  | Living alone | -0.1565 | -0.0112 | -0.1816 | 0.1019 |
|  | (0.8645) | (0.9087) | (-0.2216) | (-0.1593) |  |  | (-0.5369) | (-0.0422) | (-0.8770) | (0.3767) |
| Type of medical insurance | -0.0364 | -0.0244 | -0.0533 | 0.015 |  | Type of medical insurance | -0.0154 | 0.068 | 0.0604 | -0.071 |
|  | (-0.6594) | (-0.4237) | (-0.9771) | (0.1986) |  |  | (-0.2217) | (0.8471) | (0.9305) | (-0.7899) |
| Number of chronic diseases | -0.0265 | 0.1515^**^ | 0 | -0.0874 |  | Number of chronic diseases | -0.0234 | -0.027 | 0 | 0.0822 |
|  | (-0.6371) | (2.4209) | (.) | (-1.6266) |  |  | (-0.4204) | (-0.3804) | (.) | (1.1831) |
| Number of living children | -0.0501 | -0.1663^**^ | -0.0822 | -0.1333^*^ |  | Number of living children | 0.0267 | -0.0193 | -0.0055 | 0.0984 |
|  | (-0.6288) | (-2.5082) | (-1.6382) | (-1.8212) |  |  | (0.32) | (-0.2687) | (-0.0856) | (1.1129) |
| Pseudo R^2^ | 0.0341 | 0.0046 | 0.0226 | 0.0130 |  | Pseudo R^2^ | 0.0012 | 0.0142 | 0.0106 | 0.0093 |
| *N* | 2,961 | 3,181 | 3,190 | 3,443 |  | *N* | 907 | 1,126 | 1,080 | 1,125 |

*Notes*: This method compares the logit regression results for each year before and after matching. The coefficient values of the covariates decreased and became statistically insignificant, and pseudo R^2^ significantly decreased after matching. These results indicate that there was no systematic bias in the covariates of the two groups in different years.

*Significance levels*: ^*^ *p* < 0.05, ^**^ *p* < 0.01, ^***^ *p* < 0.001
