# Supplementary material for: Evaluating the impact of a pilot programme for home- and community-based services on long-term care needs among older adults in China
Source: PLoS One. 2024 Nov 21;19(11):e0311616. doi: 10.1371/journal.pone.0311616 (PMC11581224; doi:10.1371/journal.pone.0311616)
Supplement: S4 Table — (DOCX) [file pone.0311616.s004.docx]

**S4 Table. Heterogenous analysis with interaction term (DiD without matching)**

|  | Number of unmet ADL needs | Number of unmet IADL needs | Levels of ADL needs | Levels of IADL needs |
| --- | --- | --- | --- | --- |
|  | (1) | (2) | (3) | (4) |
| *Panel A. By the oldest old (>80 years old) or younger* | | | | |
| Policy × oldest | –0.143 | 0.198 | 0.152^*^ | 0.233 |
|  | (0.295) | (0.122) | (0.070) | (0.121) |
| Policy | 0.027 | –0.311^*^ | –0.107^**^ | –0.187^**^ |
|  | (0.212) | (0.124) | (0.035) | (0.055) |
| *Panel B. By non-agricultural/agricultural hukou* | | | | |
| Policy × agricultural hukou | 0.145 | –0.058 | –0.036 | 0.178^*^ |
|  | (0.297) | (0.091) | (0.067) | (0.080) |
| Policy | –0.172 | –0.167 | –0.029 | –0.211^**^ |
|  | (0.260) | (0.141) | (0.049) | (0.054) |
| *Panel C. By East China or other regions* | | | | |
| Policy × East | 0.164 | 0.257 | 0.139^**^ | 0.127^*^ |
|  | (0.297) | (0.184) | (0.048) | (0.058) |
| Policy | –0.127 | –0.323^***^ | –0.112^**^ | –0.156^**^ |
|  | (0.225) | (0.097) | (0.043) | (0.050) |
| *Panel D. By Urban/rural residence* | | | | |
| Policy × urban residence | 0.079 | 0.145 | –0.038 | –0.172 |
|  | (0.248) | (0.078) | (0.076) | (0.100) |
| Policy | –0.092 | –0.279^**^ | –0.029 | –0.003 |
|  | (0.236) | (0.098) | (0.067) | (0.087) |
| *Panel E. By household low-income status* | | | | |
| Policy × low income | 0.836 | –0.026 | –0.122 | –0.045 |
|  | (0.577) | (0.155) | (0.065) | (0.118) |
| Policy | –0.153 | –0.202 | –0.025 | –0.090 |
|  | (0.192) | (0.103) | (0.046) | (0.053) |
| *Panel F. By living arrangement* | | | | |
| Policy × living alone | –0.387 | 0.174 | 0.221^**^ | 0.215 |
|  | (0.246) | (0.167) | (0.079) | (0.131) |
| Policy | 0.054 | –0.248^*^ | –0.101^*^ | –0.148^**^ |
|  | (0.202) | (0.106) | (0.041) | (0.054) |
| City fixed effect | YES | YES | YES | YES |
| Year fixed effect | YES | YES | YES | YES |
| Observations | 1,120 | 2,548 | 12,363 | 12,363 |

*Notes*: Standard errors are in brackets. ADL, activities of daily living; IADL, instrumental activities of daily living.

*Significance levels*: ^*^ *p* < 0.05, ^**^ *p* < 0.01, ^***^ *p* < 0.001
