# Supplementary material for: Evaluating the impact of a pilot programme for home- and community-based services on long-term care needs among older adults in China
Source: PLoS One. 2024 Nov 21;19(11):e0311616. doi: 10.1371/journal.pone.0311616 (PMC11581224; doi:10.1371/journal.pone.0311616)
Supplement: S5 Table — (DOCX) [file pone.0311616.s005.docx]

**S5 Table. Robustness checks for programme impact (DiD without matching)**

|  | Number of unmet ADL needs | | Number of unmet IADL needs | | Levels of ADL needs | | Levels of IADL needs | |
| --- | --- | --- | --- | --- | --- | --- | --- | --- |
|  | (1) | (2) | (1) | (2) | (1) | (2) | (1) | (2) |
| Policy | –0.054 | –0.039 | –0.209^*^ | –0.218 | –0.051 | –0.054 | –0.099^*^ | –0.100^*^ |
|  | (0.202) | (0.232) | (0.104) | (0.115) | (0.040) | (0.040) | (0.047) | (0.048) |
| City fixed effect | YES | YES | YES | YES | YES | YES | YES | YES |
| Year fixed effect | YES | YES | YES | YES | YES | YES | YES | YES |
| LTCI fixed effect | YES |  | YES |  | YES |  | YES |  |
| Community fixed effect |  | YES |  | YES |  | YES |  | YES |
| Observations | 1,120 | 1,045 | 2,548 | 2,512 | 12,363 | 12,363 | 12,363 | 12,363 |
| R-squared | 0.1688 | 0.3004 | 0.1114 | 0.1951 | 0.0769 | 0.1205 | 0.1584 | 0.2013 |
|  | Number of unmet ADL needs | | Number of unmet IADL needs | | Levels of ADL needs | | Levels of IADL needs | |
|  | (3) | (4) | (3) | (4) | (3) | (4) | (3) | (4) |
| Policy | 0.001 | –0.008 | –0.185 | –0.149 | –0.040 | –0.037 | –0.082 | –0.109 |
|  | (0.208) | (0.169) | (0.105) | (0.097) | (0.042) | (0.037) | (0.050) | (0.056) |
| City fixed effect | YES | YES | YES | YES | YES | YES | YES | YES |
| Year fixed effect | YES | YES | YES | YES | YES | YES | YES | YES |
| Observations | 1,019 | 1,154 | 2,354 | 2,629 | 11,311 | 12,776 | 11,311 | 12,776 |
| R-squared | 0.1802 | 0.1725 | 0.1088 | 0.1097 | 0.0791 | 0.0779 | 0.1572 | 0.1585 |

*Notes*: Robust standard errors clustered at the city level are reported in parentheses. All regressions control for city fixed effects, year fixed effects, individual covariates (including age, sex, marital status, educational level, urban residence, low-income level, living alone, type of social medical insurance, number of chronic diseases, and number of living children), and city covariates (including the number of counties, logarithm of population size, logarithm of per capita gross domestic product, logarithm of public budgeted revenue, and proportion of older people in the total population). In addition, Model (1) considers the fixed effects of the long-term care insurance pilot policy. Model (2) considered the community fixed effect and did not adjust for urban residences because of collinearity; some communities were omitted because of collinearity. Model (3) excludes the 2018 pilot cities from the control group. Model (4) includes Beijing, Tianjin, Shanghai, and Chongqing in the treated group. ADL, activities of daily living; IADL, instrumental activities of daily living; LTCI, long-term care insurance.

*Significance level*: ^*^ *p* < 0.05
