# Supplementary figures and images for: Evaluating the impact of a pilot programme for home- and community-based services on long-term care needs among older adults in China
Source: PLoS One. 2024 Nov 21;19(11):e0311616. doi: 10.1371/journal.pone.0311616 (PMC11581224; doi:10.1371/journal.pone.0311616)

**S1 Fig. Distribution of propensity scores before and after matching.**
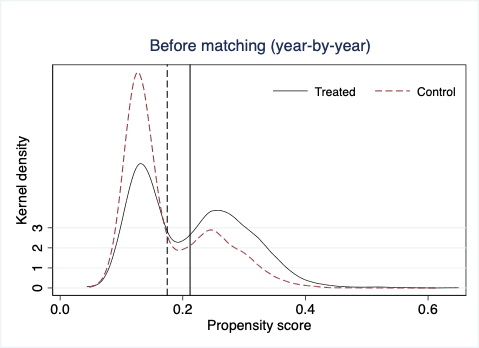

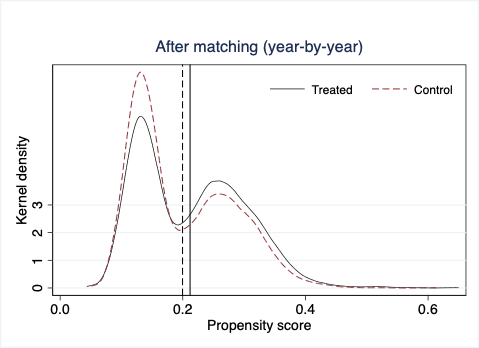

Supplement: S1 Fig — (DOCX) [file pone.0311616.s006.docx]
