# Supplementary material for: Evaluating the impact of a pilot programme for home- and community-based services on long-term care needs among older adults in China
Source: PLoS One. 2024 Nov 21;19(11):e0311616. doi: 10.1371/journal.pone.0311616 (PMC11581224; doi:10.1371/journal.pone.0311616)
Supplement: S2 Fig — (DOCX) [file pone.0311616.s007.docx]

**S2 Fig. Heterogeneity analysis with subgroups (DiD without matching)**


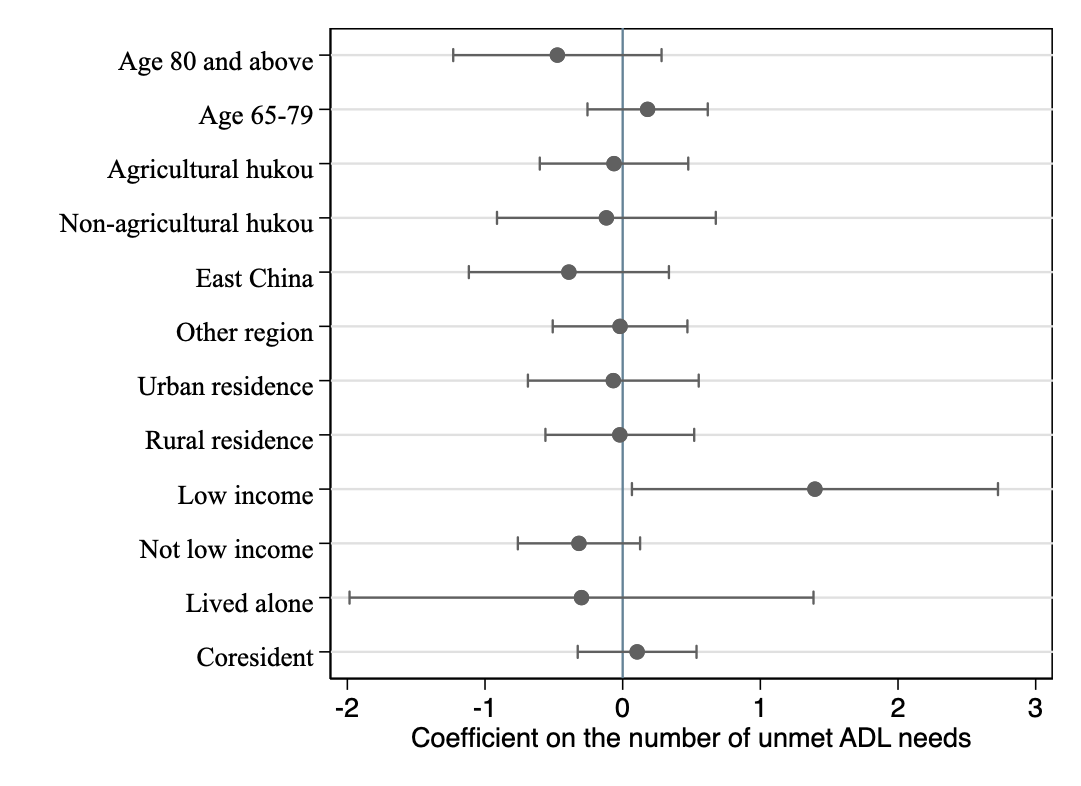

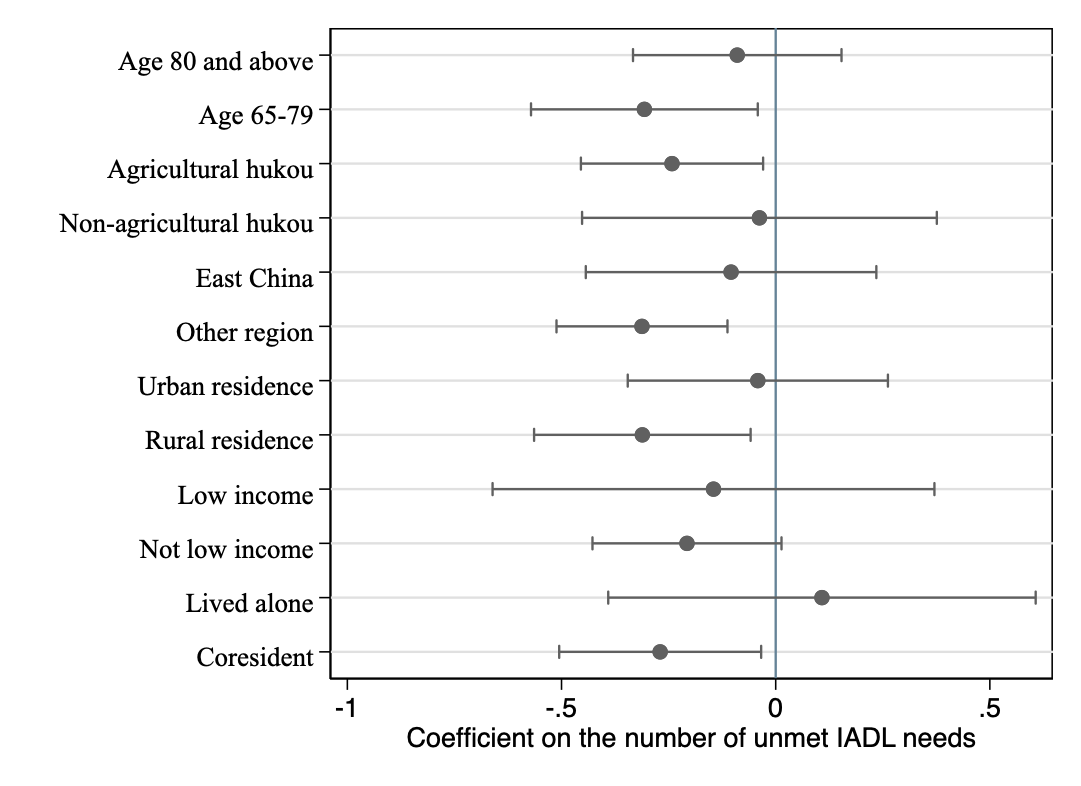


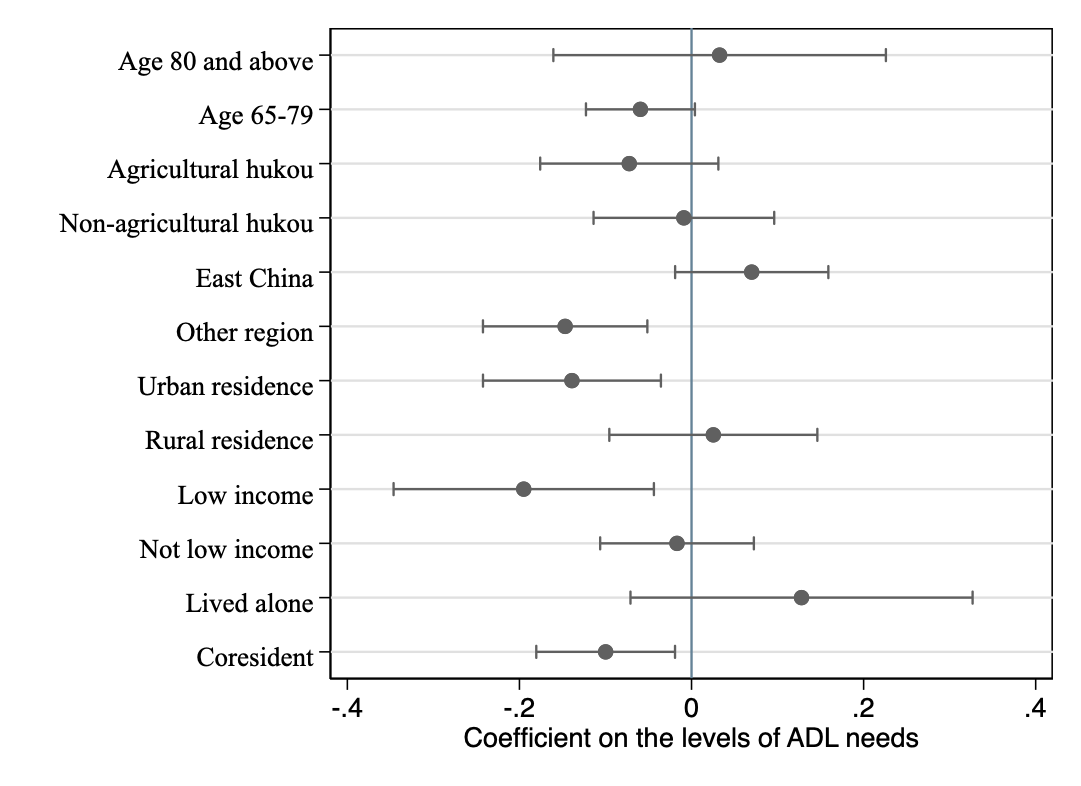

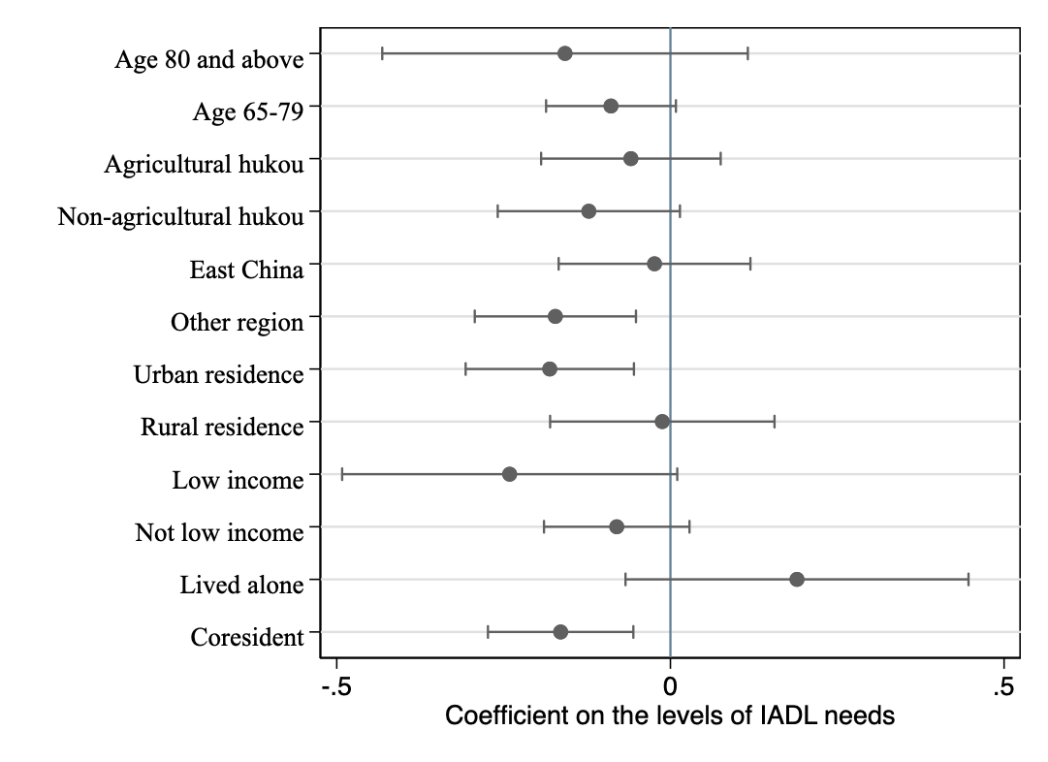


*Notes*: This figure shows the DID without matching estimates for the effect of the HCBS programme on the respective outcomes by age group, *hukou* type, geographical area, urban-rural residence, household low-income status, and living arrangement. We divided the sample into subgroups and conducted regression analysis. The dots mark the point estimates, and the lines indicate 95% confidence intervals. All regressions controlled for year fixed effects, city fixed effects, individual-level covariates, and city-by-year covariates.
