# Supplementary material for: Evaluating the impact of a pilot programme for home- and community-based services on long-term care needs among older adults in China
Source: PLoS One. 2024 Nov 21;19(11):e0311616. doi: 10.1371/journal.pone.0311616 (PMC11581224; doi:10.1371/journal.pone.0311616)
Supplement: S3 Fig — (DOCX) [file pone.0311616.s008.docx]

**S3 Fig. Parallel trend checks (without matching)**


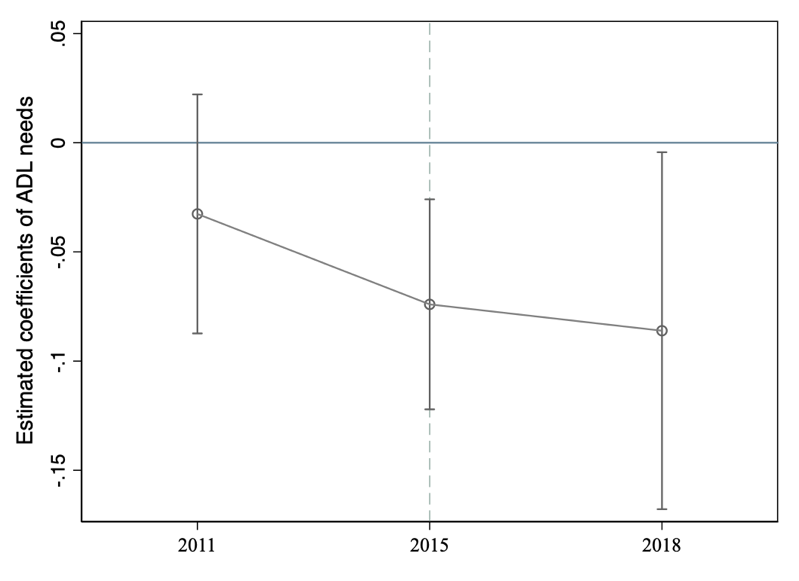


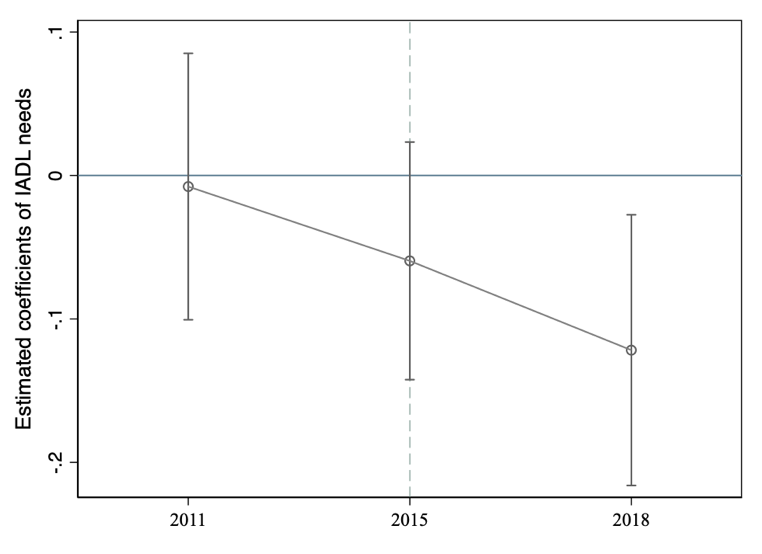


*Notes*: This figure depicts the coefficients and 95% confidence intervals of event studies for the programme’s effects on the levels of ADL and IADL needs. The year 2013 was excluded as the reference year. All regressions controlled for year fixed effects, city fixed effects, individual-level covariates, and city-by-year covariates.
